# Camera trapping expands the view into global biodiversity and its change

**DOI:** 10.1098/rstb.2022.0232

**Published:** 2023-07-17

**Authors:** Ruth Y. Oliver, Fabiola Iannarilli, Jorge Ahumada, Eric Fegraus, Nicole Flores, Roland Kays, Tanya Birch, Ajay Ranipeta, Matthew S. Rogan, Yanina V. Sica, Walter Jetz

**Affiliations:** ^1^ Center for Biodiversity and Global Change, Yale University, New Haven, CT 06520, USA; ^2^ Department of Ecology and Evolutionary Biology, Yale University, New Haven, CT 06520, USA; ^3^ Bren School of Environmental Science and Management, University of California Santa Barbara, Santa Barbara, CA 93106, USA; ^4^ Moore Center for Science, Conservation International, 2011 Crystal Drive Suite 600, Arlington, VA 22202, USA; ^5^ Department of Forestry and Environmental Resources, North Carolina State University, Raleigh, NC 27606, USA; ^6^ North Carolina Museum of Natural Sciences, Raleigh, NC 27601, USA; ^7^ Google, LLC, 1600 Amphitheatre Parkway, Mountain View, CA 94043, USA

**Keywords:** camera trap, biodiversity, data gaps, convention on biological diversity

## Abstract

Growing threats to biodiversity demand timely, detailed information on species occurrence, diversity and abundance at large scales. Camera traps (CTs), combined with computer vision models, provide an efficient method to survey species of certain taxa with high spatio-temporal resolution. We test the potential of CTs to close biodiversity knowledge gaps by comparing CT records of terrestrial mammals and birds from the recently released Wildlife Insights platform to publicly available occurrences from many observation types in the Global Biodiversity Information Facility. In locations with CTs, we found they sampled a greater number of days (mean = 133 versus 57 days) and documented additional species (mean increase of 1% of expected mammals). For species with CT data, we found CTs provided novel documentation of their ranges (93% of mammals and 48% of birds). Countries with the largest boost in data coverage were in the historically underrepresented southern hemisphere. Although embargoes increase data providers' willingness to share data, they cause a lag in data availability. Our work shows that the continued collection and mobilization of CT data, especially when combined with data sharing that supports attribution and privacy, has the potential to offer a critical lens into biodiversity.

This article is part of the theme issue ‘Detecting and attributing the causes of biodiversity change: needs, gaps and solutions’.

## 1. Introduction

Protecting species in the wake of increasing pressures from climate and land use change requires detailed, current and updatable information on species distributions and trends in space and time [1–5]. Such information is critical not only for local to regional-scale conservation efforts, but also to support international conservation efforts. At the 15th meeting of the Conference of the Parties to the UN Convention on Biological Diversity (CBD) in December of 2022 (COP15), nations adopted the Kunming-Montreal Global Biodiversity Framework. The need for equitable access to data to support established targets is so fundamental to the success of the framework overall that it is specifically recognized in Target 21 which requires that ‘best available data, information and knowledge, are accessible to decision makers, practitioners and the public’ [6].

Unfortunately, the widely documented biases in available data paints a highly incomplete picture of biodiversity [7–10]. Therefore, robust data collection and mobilization are necessary to address Target 21 and support the essential information on the status and trends in biodiversity, or essential biodiversity variables [11–14]. The Group on Earth Observations Biodiversity Observation Network (GEOBON, http://geobon.org) and many regional efforts and observation networks are aiming to support this need [15]. Despite ongoing advances in data collection and mobilization, large geographical and taxonomic biases in the availability of biodiversity data continue to persist [7–10]. Even among terrestrial vertebrates, which tend to be the best-documented taxonomic group, biodiversity data still disproportionately describes species in higher income nations and favours bird species over other taxa [8,9]. These gaps threaten to leave many ecosystems and species vulnerable, with unknown consequences for their persistence and places an undue burden on nations with more limited capacity to collect data to accurately track progress on international targets. Therefore, there is a desperate need to develop species *in situ* monitoring systems which can sustainably provide rapid and verifiable information [15].

Automated audio and visual sensors (e.g. bioacoustic sensors and camera traps (CTs)) are increasingly being deployed by ecologists to support a myriad of research questions [16–19]. These technologies have the capability to provide novel characterizations of natural systems, as they facilitate data collection at larger spatio-temporal scales than what is possible using traditional data collection techniques, such as point counts and sign surveys [20,21]. Automated sensors therefore could serve a critical role in closing existing biodiversity data gaps and improving continuity in monitoring by providing near-continuous streams of species observations. CTs, remote cameras triggered by pre-programmed schedules or motion sensors, are widely deployed because they serve many purposes and are accessible to a variety of users [22,23]. Although they may be sensitive to other taxonomic groups, current CT methodologies primarily collect information on medium- and large-sized ground-dwelling mammal and bird species, with more recent extensions to arboreal mammals [24–26]. CTs are relatively easy to deploy, provide a verifiable record by storing a digital image or video that can be reevaluated and can follow a more standardized sampling protocol than human observation which is subject to less transparent biases [23].

However, the potential of automated sensors to contribute to conservation and research applications across local to global scales is hampered by the challenges associated with processing the vast amounts of data they create and limited data sharing. Computer vision models, or machine learning approaches for classifying images, have greatly increased the efficiency of processing images by allowing for automated identification of the species present within an image [27]. While these models have improved greatly in recent years, future advancements depend on access to large collections of images to use as training examples, further emphasizing the value of data sharing [28].

The true power of automated biodiversity sensors will be realized through platforms which support the sharing and rapid translation of data into actionable knowledge. The Wildlife Insights (WI) initiative (https://wildlifeinsights.org/) was founded with this need as its core mission [23]. The WI platform deploys artificial intelligence trained on contributed data to facilitate species-level identifications of user-uploaded images from CTs, supports common analytics and enables data sharing. WI is addressing gaps in access to advanced analytical approaches by developing built-in data analysis, such as occupancy modelling. Therefore, WI is working to create a more equitable application of CTs by supporting the translation of data into actionable insights for a broad range of users to meet their individual monitoring goals.

In addition to supporting the needs of individual conservation practitioners and researchers, WI encourages and supports data sharing to create a more robust information base to assess the status of global wildlife populations. To incentivize data sharing, WI allows all users to embargo their data up to 48 months after uploading, with the possibility of extending on a case-by-case basis. Since its launch in summer 2021, WI has developed an expanding user base with *ca* 20 million wildlife records available for public download on the WI website, already constituting the largest public repository of CT data (wildlifeinsights.org as of January 2023). Although other platforms which support CT data exist, none offer the same level of controls over data sharing as WI. eMammal was a popular platform for storing and processing CT data with streamlined processes for manually identifying species. The eMammal platform is collaborating closely with WI, and all public eMammal data are now available through the WI platform. The Agouti platform (https://agouti.eu/) provides image identification backed by artificial intelligence and currently hosts *ca* 91 million images. However, none of this data is available for public download. The Labeled Image Library of Alexandria (https://lila.science) contains annotated images from many different sources, not just CTs, with the primary goal of supporting computer vision development for conservation work. Although users can download images and metadata, they do not have access to information on the location and time the image was taken, metadata on camera operability or standardized taxonomy. By lowering technical barriers for individual users working with CT data and supporting data sharing, WI is increasing the accessibility of CT data.

While still in its early phases of adoption, we sought to assess if the growing collection of CT data from WI is already filling global biodiversity information gaps by increasing data accessibility. We investigated the ways in which these CT data complement existing publicly available occurrence records from the Global Biodiversity Information Facility (GBIF). As the largest aggregator of biodiversity data across observation types, GBIF hosts a wide range of biodiversity data types, but does not currently capture the detailed metadata necessary to preserve CT data in its original form. Specifically, we broadly followed the methodology of the Species Information Index (SII) [9], which has been adopted as a component indicator for the Global Biodiversity Framework's Target 21 [6]. The index assesses annually how well available data documents actual occurrence within species potential geographical range.

**Figure 1.**
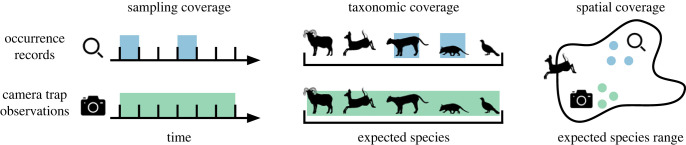
Potential for camera trapping to fill biodiversity information gaps. We assessed the potential for publicly available CT images from the WI platform to fill biodiversity information gaps by complementing existing occurrence records from various sources available through the GBIF. We tested for the complementarity of CT data by investigating whether CT (*a*) sampled locations for a greater duration, (*b*) sampled a broader array of species in a given location and (*c*) sampled unique portions of species' ranges. (Online version in colour.)

We compared coverage with and without CT data under this indicator framework. We hypothesized that CT data may complement the coverage of existing biodiversity records in three ways (figure 1). First, cameras automatically collect repeated observations and therefore may sample more days per year in a given location as compared to other, more idiosyncratic sampling techniques. Second, cameras collect information on many species, so that, in a given location, CT data may observe a larger proportion of species expected to occur there compared to other data collection techniques, especially difficult to detect or elusive species. Third, cameras may more easily be deployed in remote locations compared to other sampling techniques and therefore may provide information about portions of species' ranges not covered by other data sources.

## 2. Methods

### (a) Species occurrence data

We compared trends in data collection and coverage of spatio-temporal biodiversity records from the WI platform to those aggregated by the GBIF for mammal and bird species [29]. GBIF currently aggregates records collected by CTs as a form of ‘machine observations’, but it does not distinguish CTs from other sensor types such as acoustic monitors and animal tracking devices. Therefore, it is impossible to easily filter out CT data from GBIF records using the current metadata format. WI data are not currently exported to GBIF. Thus, we include all GBIF records in this analysis without disaggregating CT observations. Doing so likely biases our results towards undervaluing the role of CT data. Importantly, the metadata associated with GBIF records does not rigorously record sampling protocols or effort and absences are rarely reported by users.

**Figure 2.**
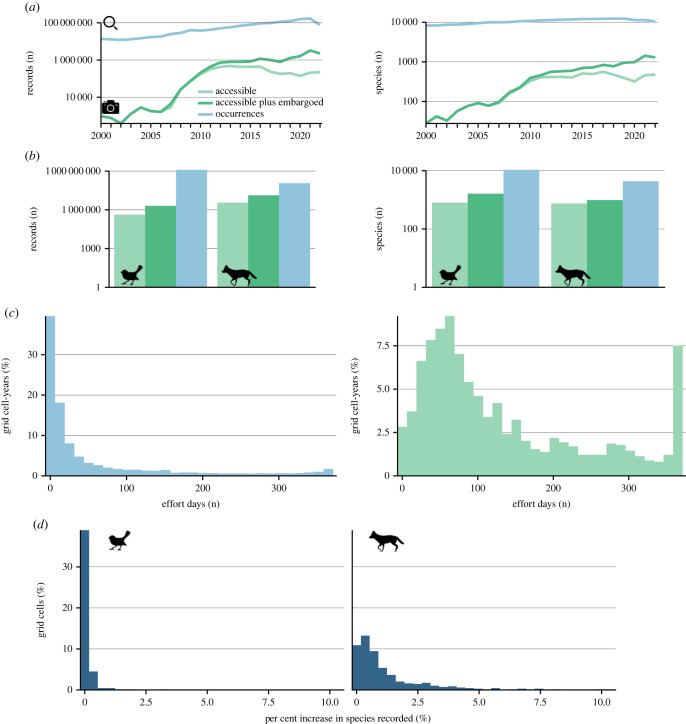
Global biodiversity data collection. (*a*) Annual number of records and species collected from each dataset. (*b*) Total number of records and species available as occurrence records (blue) and CT images (green) (2000–2022). (*a*,*b*) Number of records and species in the WI platform are reported for data which is not under embargo (light green) and all data, including datasets currently under embargo (dark green). See the WI Terms of Service for full details (https://app.wildlifeinsights.org/terms-of-service). (*c*) Number of unique days per year with point occurrences of birds or mammals (left) and active CT deployments (right) within 110 × 110 km grid cells (2010–2022). (*d*) Difference in the per cent of expected species per 110 x 110 km grid cell that was recorded by WI data and not sampled with occurrence records from GBIF. (Online version in colour.)

As the main source of publicly available CT data, we consider WI to be the most comprehensive dataset to assess data coverage for CT data. GBIF and WI data were downloaded on 01 June 2022 and 26 November 2022, respectively. CTs routinely take repeated images of the same individual animal. WI has the option to consider images either individually or as part of the same ‘sequence’ if they are taken at the same location less than 1 min apart. Out of *ca* 63 million images and 3 million sequences hosted in the WI platform as of November 2022, we selected all records of wild mammal and bird species for which the computer vision-based classification was reviewed by users. This resulted in a total of *ca* 15.3 million records (14.1 million images and 1.1 million sequences) of wild mammal and bird species collected between 1 January 2000 and 23 November 2022 (616 647 active trap days), 4.0 million of which were not currently under embargo and resulted in 128 161 unique species occurrences when aggregated by location and year [11,30–418]. It is important to note that WI allows users to automatically embargo data that are part of ongoing data collection for up to 48 months. Under this embargo, data are protected from being included in derived analytical products in peer-reviewed publications (for latest WI Terms of Service, https://app.wildlifeinsights.org/terms-of-service). We report the total number of records and species in both the non-embargoed and embargoed datasets (figure 2*a*). However, all subsequent analyses are based solely on non-embargoed records from WI data.

Records from both datasets were taxonomically harmonized using synonym lists built for this purpose by Map of Life and collaborators (https://mol.org/). Scientific names were considered taxonomically valid if they could be linked to the established authoritative name based on known synonyms. We used species delimitations for mammals based on the Mammal Diversity Database [419] and birds based on eBird/Clements Checklist of Birds of the World [420]. Custom-built synonym lists compiled potential synonyms and typographical variants to these names from additional sources [421–430]. All species names in WI were harmonized to taxonomic authorities and less than 0.1% of GBIF records were unable to be resolved.

### (b) Sampling coverage

To highlight the differences in sampling coverage between datasets, we estimated the number of unique days of data collection within 110 × 110 km grid cells. Because GBIF data do not uniformly include sampling effort, we instead determined the number of days in a location with records. For GBIF data, we spatially intersected all bird and mammal records from 2010 to 2022 with the equal area grid. We then calculated the number of unique days on which records were collected per year within each grid cell. For WI data, we spatially intersected locations of CT deployments with the same equal area grid. We then determined the number of unique days with active deployments per year within each grid cell. In this case, we define a deployment as the placement of a camera at a location for a specific period of time. All data providers to WI are required to report the start and end dates for which a camera was active. We acknowledge that in the case of GBIF data, this approach may underestimate the number of days on which sampling occurred by not including days on which no species were detected or in cases where the number of sampling days are not reported. However, our goal was to demonstrate how the current structure of both datasets supports interpretation and this information is currently not readily available to GBIF users.

### (c) Taxonomic coverage

To investigate if CT data samples a greater proportion of species in a given location relative to other data collection techniques, we estimated the species expected to be present within 110 × 110 km grid cells based on expert range maps. Mammal ranges were estimated based on expert-based range maps from Marsh *et al.* [431] and bird ranges from Jetz *et al.* [430]. Species ranges were coarsened to 110 × 110 km grid cells to minimize false presences [430,432,433]. Spatio-temporal species observations from 2010 to 2022 were intersected with the same equal area grid to determine the number of species observed in each grid cell. We computed the percentage of expected species observed based on (i) GBIF records and (ii) the combined GBIF and WI records. We then computed the difference in per cent of species observed when WI records were added to GBIF records. For this analysis, WI records were coarsened to the annual scale; as such, the difference between images and sequence records was no longer relevant and a record was considered as any detection of a species at a certain location and year.

### (d) Spatial coverage

Spatial biodiversity data coverage was assessed using the SII [9]. The SII estimates the proportion of a species' range with observations in a given year. For each species, their expected range was intersected with a 110 × 110 km equal area grid to determine the number of grid cells where they are expected to occur. Spatio-temporal species observations were intersected with the same equal area grid to determine the number of grid cells with observations in a given year. The SII is then computed as the proportion of expected occupied grid cells with observations in a given year. For example, a value of 0.5 indicates that 50% of grid cells in which a species is expected to occur had data. It is important to note that values are not necessarily expected to approach the maximum value of 1 as this would constitute complete coverage of species range in a single year. Even for well-documented species, such as birds, the global mean in annual data coverage is less than 20% [9].

Full details of the SII methodology can be found in Oliver *et al.* [9]. In this study, the SII was computed for species individually and aggregated at the national level. National SII aggregates the species-level SII by averaging species values across all species expected in a country considering only the portion of the range within national borders. National boundaries were determined based on the Database of Global Administrative Areas (GADM version 3.6, gadm.org).

**Figure 3.**
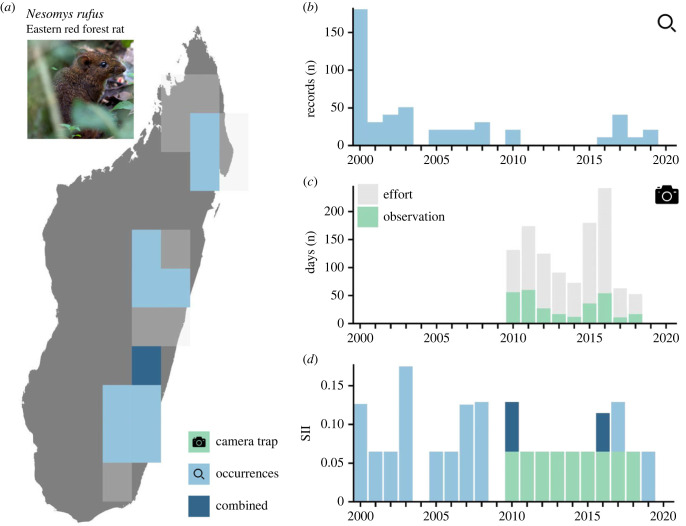
Empirical example of data collection approaches for the Eastern red forest rat (*Nesomys rufus*). (*a*) Expected range and biodiversity observations of the Eastern red forest rat in Madagascar. Grid cells (110 × 110 km) expected to be occupied are shown in light grey. Grid cells with observations from point occurrences shown in light blue. Grid cells with observations from both point occurrences and CT data shown in dark blue. (*b*) Number of records collected per year as point occurrences. (*c*) Number of days of CT data collection. Light grey indicates the number of days per year of active data collection within deployments in the species’ range. Green indicates the number of days with CT observations. (*d*) The SII [9] computed based on point occurrences (light blue), CT data (green) and both data types (dark blue). (Online version in colour.)

We demonstrate the SII and how we assess the complementarity of WI and GBIF data for a single species, the Eastern red forest rat (*Nesomys rufus*) (figure 3).

Species and national SII were computed for terrestrial mammals and birds independently based on (i) GBIF records and (ii) the combined GBIF and WI datasets. SII values were averaged over the previous 12 years (2010–2022). The boost in data coverage provided by incorporating WI records was determined by the per cent difference in mean SII computed based solely on GBIF records and the combination of GBIF and WI records. For this analysis, WI records were coarsened to the annual scale; as such, the difference between images and sequence records was no longer relevant and a record was considered as any detection of a species at a certain location and year.

## 3. Results

### (a) General data summary

Comparing raw spatio-temporal species records between sources, annual data collection differed greatly between datasources. From 2000 to 2022, data collection for birds and mammals averaged *ca* 60 million records and 10 583 species per year in GBIF and *ca* 420 thousand records and 385 species per year in WI (figure 2*a*). GBIF contained *ca* 116 million records from 4338 mammal species and *ca* 1.25 billion records from 10 736 bird species (figure 2*b*). As of November 2022 WI contained *ca* 13.3 million records from 977 mammal species and *ca* 2 million records from 1628 bird species. Of these *ca* 3.5 million records from 748 mammal species and *ca* 400 thousand were not subject to embargo (figure 2*b*). Total annual data collection in GBIF peaked in 2021 (2014 for mammals and 2021 for birds) (figure 2*a*; electronic supplementary material, figure S1a). Total annual data collection within WI was highest in 2021 and highest for non-embargoed data in 2013 (2016 from mammals and 2013 for birds) (figure 2*a*; electronic supplementary material, figure S1a). Taxonomic coverage of birds and mammals peaked in GBIF in 2019, among all records in WI in 2021, and among public records in WI in 2017 (2013 for birds and 2016 for mammals) (figure 2*a*; electronic supplementary material, figure S1b). Both datasets showed a declining number of records in the previous 2 years. At time of submission, only records stored in GBIF as of 1 June 2022 were included in analyses, thus the total data collection for 2022 are incomplete, and it is very likely that many records collected within 2021 have yet to be fully mobilized. The decline in records is especially apparent for the publicly available data within WI. This is likely due to users’ ability to embargo data recently uploaded to the platform for up to 48 months.

### (b) Sampling coverage

The number of unique days of data collection within 110 × 110 km grid cells during the period 2010–2021 was significantly higher from WI data (mean = 133 days, median = 92 days) compared with GBIF data (mean = 57 days, median = 12 days) (figure 2*c*; *t*-test: *t* = −24.47, d.f. = 1265.4, *p* < 0.001). From GBIF data, 65.3% of grid cell-years had less than or equal to 30 days of data collection, compared to 12% from WI data. By contrast, 19.1% of grid cell-years had over 100 days of data collection from GBIF data, compared to 46.6% from WI data.

### (c) Taxonomic coverage

WI data provided records of mammal species not found in GBIF in 92% of the 380 grid cells where it was present. Where WI data for mammals was collected, it recorded an additional 1.2% of species on average compared to GBIF data and a maximum increase of 10.3% additional species. WI data provided records of bird species not found in GBIF in 34% of the 308 grid cells where it was present. Where WI data for birds were collected, it recorded an additional 0.8% of species on average compared to GBIF data and a maximum increase of 3.4% additional species.

### (d) Species example

Of the 19 grid cells expected to be occupied by the Eastern red forest rat (*Nesomys rufus*) in Madagascar, 10 contained GBIF records and 1 also contained WI records over the period 2000–2022 (figure 3*a*). Data collection from GBIF varied considerably between years. From 2000 to 2022, on average 23.3 GBIF records were collected per year (figure 3*b*). Although data collection from CTs occurred over a shorter timespan than GBIF data collection, CTs more consistently collected observations. Over the same period, there were on average 40 days per year of active CT deployments and on average 32 days per year with observation of the Eastern red forest rat (figure 3*c*). The two data sources were complementary in providing consistent coverage of the Eastern red forest rat's range. Mean data coverage, as estimated by the SII, was 0.057 from GBIF data, 0.028 from WI data and 0.078 when both datasets were combined (figure 3*d*).

### (e) Species spatial data coverage

**Figure 4.**
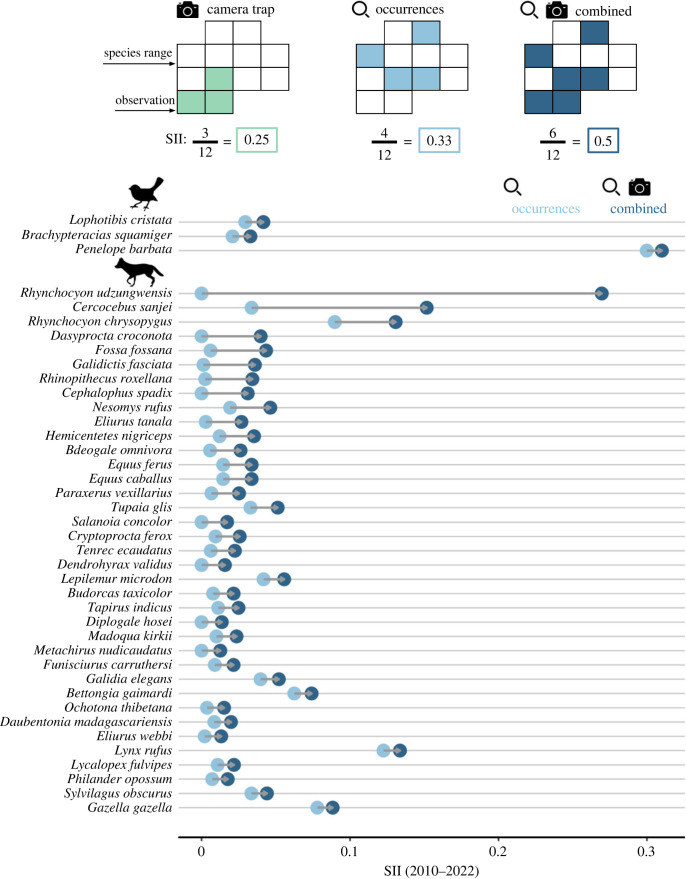
Species boost in data coverage from camera trapping. (Top panel) Schematic explanation of the SII. The SII estimates the proportion of grid cells within a species' expected range with observations. For this hypothetical species, data coverage is increased when CT data and point occurrences are combined. (Bottom panel) Mean data coverage over the previous decade (2010–2022) for species with a greater than 0.01 increase in data coverage with the addition of CT data. Light blue dots represent SII based solely on occurrence data. Dark blue dots represent SII based on the combination of occurrence and CT data. (Online version in colour.)

The addition of CT data was more important for improving data coverage for mammals than bird species. WI data increased mean SII (2010–2022) for 655 out of 704 mammal species and 304 out of 627 bird species (93% and 48% of species with CT data) relative to SII based solely on GBIF data. For species with WI data, CT data covered less than an additional 0.1% on average of bird species ranges and 0.3% on average of mammal species ranges. For 37 mammal species and 3 bird species, SSII increased by at least 0.01 (i.e. an additional 1% of the species range covered) (figure 4).

### (f) National spatial data coverage

**Figure 5.**
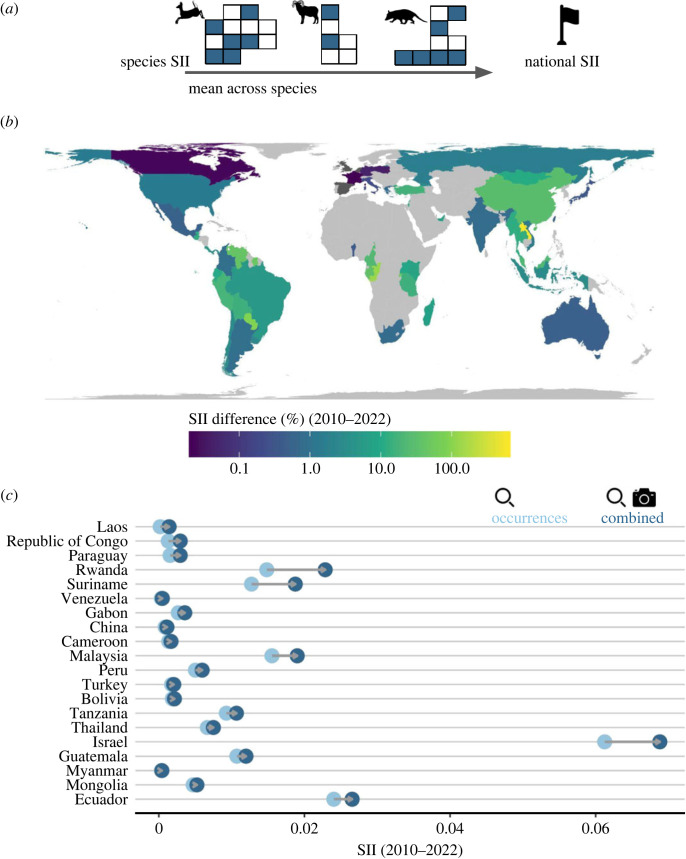
National boost in data coverage from camera trapping. (*a*) Schematic explanation of the Species Information Index (SII) at the national scale. National SII values are estimated as the mean SII values across species expected to occur within a given nation, considering only the portion of the species' ranges within national borders. (*b*) Per cent difference in mean data coverage over the previous decade (2010–2022) with the addition of CT data to occurrence data for mammal species. Light grey indicates countries with currently no data in WI. Dark grey indicates countries with CT data, but no increase in data coverage. (*c*) Mean data coverage over the previous decade (2010–2022) for nations with greater than 10% increase in data coverage with the addition of CT data. Light blue dots represent SII based solely on occurrence data. Dark blue dots represent SII based on the combination of CT and occurrence data. Nations listed top to bottom based on per cent increase in data coverage. No nations had an increase in data coverage for bird species greater than 2%. (Online version in colour.)

For mammal species, WI data increased mean national SII (2010–2022) for 49 of 53 nations where data were present (figure 5*a*). National SII increased by greater than 10% for 20 countries with a mean increase of 7% (figure 5*b*). For bird species, WI data increased mean national SII (2010–2022) for 33 of 46 nations where data were present. The mean increase was less than 1% and no nations had increases greater than 10%.

## 4. Discussion

While the diverse data stores available through GBIF represent a substantially higher volume of records than the current collection of CT records in WI, we already find evidence that CT data complements other biodiversity data sources at the global scale. CT data expanded both the amount of time locations were sampled and the number of species recorded. Despite large differences in data volume, we found that in the locations where CTs were deployed, they recorded species not documented by other data sources, particularly for mammals. Although CTs may be deployed to monitor a specific species, they record information of all species for which they capture an image. Cameras may also be less obtrusive than other data collection techniques and thus better able to detect elusive species. CT studies provided much more consistent sampling in the locations where they were deployed. Approximately 10% of location-years where CTs were present had active deployments year round. This stands in stark contrast with the sampling coverage from publicly available occurrence records, which in the majority of cases provided less than a month of total sampling per year. We acknowledge that our estimates of sampling intensity likely underestimate the true intensity of GBIF data; however, our intention was to highlight the information that downstream users readily infer from the current data structure. While fairly simplistic in its formulation, this comparison gives strong support to repeated calls to leverage automated sensors for more reliable, consistent, long-term monitoring [19,21]. Repeated sampling of the same locations over time, as shown for CT studies, will support a more rigorous quantification of biodiversity change by allowing for the identification of changes in community structure and potential local extinctions and range shifts.

For hundreds of species, the addition of CT data provided observations of their expected ranges not previously covered by publicly available occurrence records. The majority of these species were mammals, which also tended to see larger boosts to their spatial data coverage. Species for which CT data was most important represented a diverse set of small-to-medium bodied mammals including rodents, carnivores, ungulates and primates. These results suggest the potential for CTs to fill taxonomic data gaps by providing information on less well-studied taxa. In general, larger bodied mammals, particularly charismatic carnivores, receive greater monitoring attention, thereby leaving less charismatic species comparatively poorly understood [434,435]. These large carnivores are also often the target of many CT studies, but the ability of cameras to collect information simultaneously on many species lead to collecting information on less-studied taxa. Traditionally, CT data have been better suited to detecting medium and large-bodied, ground-dwelling mammals because of common deployment techniques [24]. However, as deployment in tree canopies grows more popular, cameras are increasingly detecting arboreal species [26]. In addition, CTs may be better suited to studying cryptic or rare species that may go undetected by human observers [436–438], and to sample remote areas for relatively long periods. Importantly, CT data uniquely serve as a verifiable record compared to human observations by allowing for re-examination [23].

Scaling up from individual species, the importance of camera trapping was evident at the national scale by filling biodiversity information gaps for previously unsampled regions or species. Nearly all nations with CT records increased in data coverage for mammals and a majority increased in coverage for bird species. Boosts in data coverage were particularly strong for nations in South America as well as portions of Africa and eastern Asia, which tend to be poorly represented in global biodiversity data stores [8,9]. Therefore, we find evidence that CT data may help close long-standing geographical biases in biodiversity information at least for mammals and ground-dwelling birds. Doing so is critical to achieve international targets such as Target 21 of the CBD's Global Biodiversity Framework which requires nations to develop an improved, shared knowledge base. This task is particularly urgent for tropical nations which hold disproportionately high biodiversity despite many species often remaining poorly documented. Based on their demonstrated potential to fill data gaps, CTs could serve as a critical piece in establishing long-term semi-automated biodiversity monitoring in areas relatively under-sampled [18,439].

Unsurprisingly, we find that the contribution of CT data in closing information spatial and taxonomic gaps was more substantial for mammal species than for bird species. This is likely due to two main factors. First, the WI data available for this analysis contained more records of mammals than birds, despite addressing a similar number of species. In general, camera trapping studies tend to be more focused towards mammal species than birds. Additionally, the majority of studies deploy cameras near the ground, where relatively fewer bird species are active, and with settings and placement of the devices aimed at detecting large-bodied species. Second, data collection for bird species within GBIF vastly outpaces that for mammal species in part due to the widespread popularity of taxon-specific citizen science platforms such as eBird [9]. Given the particularly comprehensive documentation of bird species already available through other data sources, it would be challenging for new datasets to contribute complementary information at this scale. However, the increasing number of camera trapping efforts focusing on sampling within the tree canopy might provide more information on bird species (and other taxa) in the future [26]. For example, CTs have even been shown to be effective in monitoring insect–plant interactions [440]. Future integration with other sensor types, such as audio recorders, will greatly increase the taxonomic scope of CT studies, by detecting a range of vocal taxa, including birds, amphibians and insects. Passive acoustic monitoring is increasingly being used to monitor a diverse array of vocal species and platforms have developed to support data processing and sharing, such as Rainforest Connection's Arbimon, as well as assist in creating machine learning methods, such as the Earth Species Project [441]. Co-deployment of cameras and audio sensors has the potential to expand the scope of both techniques by detecting complementary species [19].

Our study provides an extension of the SII, a component indicator adopted for Target 21 of the Kunming-Montreal Global Biodiversity Framework (https://www.cbd.int/doc/decisions/cop-15/cop-15-dec-05-en.pdf) to explore the contribution of specific data types and sources. We find that methodology allows identifying complementary values of distinct data contributions, and we specifically uncover such a role for CT data. CT data may provide additional benefits above more traditional occurrence and survey data, for example by providing repeat observations at the same site (e.g. weeks of sampling). However, our analysis of the number of days in a region that received sampling provides an indication of the potential for repeated observations. Information about sampling effort can be fed into statistical methods that leverage repeated observations to correct for imperfect detection to estimate population size and distribution [442]. Detection/non-detection data from CT studies are largely used to estimate probability of occupancy and species–habitat relationships for single-species and communities within occupancy modelling frameworks [22,443,444]. For species with individually distinctive markings, CT data are used to estimate population density and abundance via capture–recapture approaches [445,446]. For unmarked species, CT data enable estimating abundance through methodologies based on several data formats, from detection/non-detection or counts data [447,448], to partial identification [449], to information about the time or location of the observations [450,451] and species characteristics [52,452]. Additionally, CT data are increasingly used to explore species' behavioural responses to anthropogenic stressors [453,454] and species interactions [455,456]. Neither of these key aspects of camera trapping data is captured through SII.

It is important to note that GBIF does include data from other camera trapping studies as well as data from long-term repeated surveys and monitoring efforts. However, the information on the methodology implemented, survey effort and scope is currently captured in an unstructured manner and does not allow for disaggregating CT observations easily. Depending on the purpose, camera trappers may use a variety of attractants (visual, olfactory and food/reward-based) to intentionally attract species of interest, which is identified in the survey metadata [443]. Efforts are ongoing to address this issue, including an extension to Darwin Core standard data model [457] currently under development to improve the capture and interoperability of ecological data [458]. GBIF is currently in the process of adopting the Camera Trap Data Package (CameraDP; https://tdwg.github.io/camtrap-dp/) which is a community-developed data exchange format for CT data. Adoption of these data models would allow better reporting of CT data including digital products, survey design, targeted species and species absences and allow CT data to maintain its original structure within GBIF. It is important to recognize that each platform serves different roles. The WI platform enables users to generate insights from their data for local or regional applications as well as contribute data to the shared public repository with plans to publish (or share) in GBIF. As our work demonstrates, CT data already constitutes a valuable contribution to global data aggregators, such as GBIF, which will surely continue as efforts to create interoperability between platforms that both promote data sharing while preserving the unique characteristics of different data types continue.

Our results represent the contribution of WI to global biodiversity data coverage of terrestrial birds and mammals, not camera trapping as a whole, as there are many millions or billions of additional records in private CT databases. Our results also highlight the gains in global biodiversity data coverage from increasing accessibility of CT data, not necessarily increasing data collection. Many parts of the world remain understudied in CT studies. As is the case for biodiversity monitoring in general, CT data collection in Africa remains much lower than on other continents and within Africa a large focus is concentrated in South Africa [459,460]. As tools to process CT data become more accessible, we anticipate data collection efforts to grow. For example, WI is developing a desktop client which will allow users to take advantage of WI functionality without access to an Internet connection. As the platform, and associated data, continues to grow it will be critical to track progress on how CT data complement and support global biodiversity monitoring for terrestrial vertebrates. This exercise already shows the power of centralized repository for CT data for moving forward ecological, biodiversity and conservation research, as it has already been the case for other platforms developed to host specific data types (e.g. Movebank and GenBank). Our results highlight the potential to reduce long-standing biodiversity data gaps by leveraging existing CT data.

The long-term potential of CTs and other automated sensors to contribute to international conservation policy will depend on carefully balancing the needs of data collectors with those of the broader community to ensure benefits are equitably shared. In creating data sharing terms, WI engaged closely with protected area managers, government agencies, researchers and NGOs to understand the requirements for them to comfortably share data on the platform. The option to temporarily embargo data was requested from data providers to maintain private access to data while carrying out initial conservation and research activities. This option incentivizes data owners to share data by providing access to data management tools during periods of active research, while ensuring the data are available for future public use.

Twenty-six per cent of data in WI are currently publicly available. Given the recent public launch of WI, we expect the proportion of public data to grow as embargoes expire in coming years. In the interim, WI provides tools to discover embargoed data and contact data owners to initiate collaboration.

For data that is shared publicly, data owners receive notifications whenever their data are downloaded and WI provides citations for each dataset to be used in publications. Although shared data is equally available to the public via the WI website, the scientific and conservation community must continue to provide equitable access to technical training for all users to use this data to inform global-scale insights.

Our results indicate that the unique aspects of CTs and their deployment provide expanded understanding of terrestrial biodiversity. By demonstrating that a growing repository of public CT data already samples (i) more consistently through time, (ii) a broader array of species and (iii) unique portions of species’ ranges, we show how CTs may be a critical tool in developing a global biodiversity monitoring system [15,18]. The continued deployment of automated biodiversity sensors paired with increased capacity building and robust funding mechanisms could help democratize access to the robust information base necessary as nations are asked to track progress on achieving international conservation goals.

## Data Availability

All GBIF records are publicly available with the associated DOI [29]. All WI records are available directly from their source. All WI data are protected by the WI Terms of Use. The majority of WI records used in this analysis are available for public download. A small minority of WI data used in this publication are not publicly shared, but the names and ARK links of all studies have been provided in the references. Supplementary material is available online [461].
